# Synggen: fast and data-driven generation of synthetic heterogeneous NGS cancer data

**DOI:** 10.1093/bioinformatics/btac792

**Published:** 2022-12-09

**Authors:** Riccardo Scandino, Federico Calabrese, Alessandro Romanel

**Affiliations:** Department of Cellular, Computational and Integrative Biology (CIBIO), University of Trento, Trento 38123, Italy; Department of Cellular, Computational and Integrative Biology (CIBIO), University of Trento, Trento 38123, Italy; Department of Cellular, Computational and Integrative Biology (CIBIO), University of Trento, Trento 38123, Italy

## Abstract

**Summary:**

Whole-exome and targeted sequencing are widely utilized both in translational cancer genomics and in the setting of precision medicine. The benchmarking of computational methods and tools that are in continuous development is fundamental for the correct interpretation of somatic genomic profiling results. To this aim we developed synggen, a tool for the fast generation of large-scale realistic and heterogeneous cancer whole-exome and targeted sequencing synthetic datasets, which enables the incorporation of phased germline single nucleotide polymorphisms and complex allele-specific somatic genomic events. Synggen performances and effectiveness in generating synthetic cancer data are shown across different scenarios and considering different platforms with distinct characteristics.

**Availability and implementation:**

synggen is freely available at https://bitbucket.org/CibioBCG/synggen/.

**Supplementary information:**

Supplementary data are available at *Bioinformatics* online.

## 1 Introduction

The interrogation of next-generation sequencing (NGS), principally whole-exome (WES) and targeted sequencing (TS), is rapidly becoming a preferred approach for the exploration of large tissue and liquid biopsy-based cohorts, especially in the context of precision medicine. In this setting, a precise benchmarking of the large collection of computational tools that are continuously developed is fundamental for the correct interpretation of somatic genomic profiling results. Although many simulators of synthetic cancer genomes and NGS data have been developed in the last years (Peng *et al.*, 2019), only a few of them focus on WES and TS data and most of them either require the generation and merge of intermediate large FASTA files (Tanner *et al.*, 2019), incorporate random genomic events (Semeraro *et al.*, 2018) or do not allow to incorporate complex somatic cancer-specific patterns (Stephens *et al.*, 2016). The generation of large-scale benchmarking datasets that are able to capture cancer-specific heterogeneous scenarios together with NGS platform-specific characteristics is hence still hindered, limiting the broad applicability of synthetic NGS-based computational tool benchmarking. To overcome these limitations, we developed synggen, a tool that enables a fast and scalable generation of platform-specific cancer and matched control WES and TS synthetic data, incorporating phased germline polymorphisms together with complex and allele-specific cancer genomic events.

## 2 Approach

Synggen is a tool written in C programming language to generate synthetic NGS files, in the form of WES or TS experiments, representing heterogeneous cancer genomes and matched controls. The tool provides two execution modes which allow to (i) exploit a set of control (non-cancer) NGS sequencing files (BAM format) to generate three *reference models* capturing a collection of data summary statistics; and (ii) combine these reference models and a set of user-specified germline and somatic genomic profiles to create synthetic sequencing files (FASTQ format).

Reference models are built across a single or a collection of WES or TS sequencing control samples, profiled with the same platform, using a fast and efficient multi-threaded cumulative pileup strategy based on (Valentini *et al.*, 2019). Specifically, synggen generates: (i) a *Read Depth Model* (*RDM*), which measures the average intra-sample depth of coverage variability by calculating the probability, across all input files, of observing sequencing reads at any captured genomic region and position; only reads aligning at the lowest genomic coordinate are considered when paired-end data is used; (ii) a *Quality Model* (*QM*), which measures the distribution of base qualities across sequencing read positions; (iii) *Position-Based Error* (*PBE*), which measures for each captured genomic position [not representing a common single nucleotide polymorphism (SNP)] the probability of observing platform-specific systematic errors supported by high quality reads and bases (Casiraghi *et al.*, 2020). When paired-end data are used to generate the reference models, insert size statistics are also computed and embedded in the RDM model.

Exploiting the created reference models, synggen allows to directly generate platform-specific cancer and matched control NGS files by incorporating in the simulated genomes a user-specified list of germline-phased SNPs and user-specified lists of somatic *allele-specific* copy number alterations (CNAs) and point mutations (PMs). Phased SNPs are used to realistically represent the genetic background of specific individuals and to generate both cancer and matched control samples. Somatic CNAs are defined as allele-specific copy numbers (pairs of values specifying the number of copies for each allele) and are incorporated across all affected WES/TS captured regions to modulate the RDM distribution and to adjust SNP allelic fractions. Of note, both overlapping and nested somatic copy number alterations can be represented (see Supplementary Methods for details). Point mutations are incorporated by specifying the affected allele and indicating the number of allele copies (in case copy number alterations are present) carrying the specific point mutation. When generating cancer genomes NGS files, CNAs and PMs are defined specifying also alteration-specific clonality values, which are used to adjust the fraction at which they are incorporated in the data. A global tumor content value is also specified by the user to adjust the fraction at which all somatic events are incorporated. Provided all these information, synggen uses an efficient multi-threaded approach to generate a specified number of synthetic sequencing reads implementing for each of them the following steps: (i) sample a genomic region and position from the RDM distribution; (ii) Generate the corresponding read sequence provided a specific read length; (iii) randomly choose one of the two alleles, sampling from a probability distribution that reflects the number of region allele-specific copies; (iv) incorporate all SNPs spanning the read (if any); (v) Generate base qualities and errors sampling first from the PBE (for all positions there annotated) and then from the QM for all the remaining read bases; (vi) In case of cancer samples, incorporate PMs if any; and (vii) Choose the strand considering the strand bias (default 0.5). When paired-end data is considered, two reads are generated in Step 2 using an insert size sampled from a normal distribution with mean and standard deviation as specified in the RDM model; Steps from 3 to 7 are executed for both reads. Details are available in the Supplementary Methods.

## 3 Results

We developed synggen to allow efficient and scalable generation of cancer and matched control targeted NGS synthetic data. Compared to previous methods (Supplementary Table S1), synggen is the only simulator providing built-in generation of platform-specific WES- or TS-based NGS reads, reproducing hence sequencing characteristics of real data and incorporating germline and somatic variants without the need of intermediate FASTA files. Differently from most available simulators, synggen allows to input specific lists of germline variants and somatic genomic events, including phased germline SNPs and somatic allele-specific CNAs and PMs, together with local and global parameters including the clonality of somatic events and the overall sample tumor content, allowing for the emulation of varied and realistic cancer- and patient-specific data across the different multi-subclones composition, tumor purity, aneuploidy and tumor evolution scenarios. Our approach allows hence for the generation of large-scale bulk WES and TS datasets, including multi-regional and longitudinal tissue sequencing datasets and liquid biopsy cfDNA sequencing datasets.

**Fig. 1.**
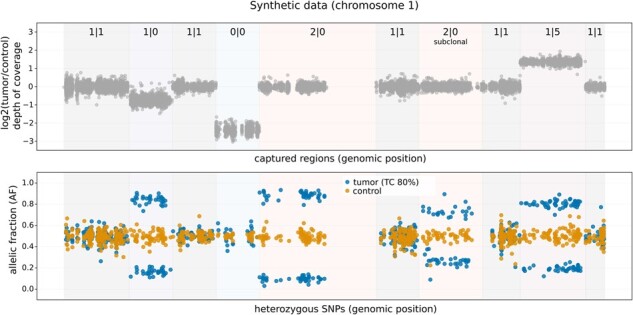
Examples of somatic allele-specific CNAs. (Top) log2(tumor/control) of regions’ average depth of coverage. (Bottom) allelic fraction (AF) of heterozygous SNPs in tumor and matched control synthetic samples. TC, tumor content

To test the performances of synggen, we generated synthetic WES and TS samples and measured the computational time required for reference models’ construction and sequencing reads generation. The construction of reference models was tested for an increasing number of input samples selected among breast cancer control (non-tumor) samples (BAM files) available from The Cancer Genome Atlas dataset, focusing on patients having high (>80%) tumor content WES (Sure Select All Exome v3) cancer tissue samples. Generation of synthetic NGS cancer data was instead tested for increasing number of sequencing reads produced. A cancer sample was generated with tumor content at 80% and incorporating patient-specific germline SNPs and patient-specific somatic CNAs and PMs (details in the Supplementary Methods). The overall reference models’ construction time using one input sample on a HPE Proliant DL560 server and exploiting 16 cores took approximately 2.5 min for the WES sample and 1 second for the TS sample (Supplementary Fig. S1). Using the same running configuration, 56 s and 1 min were instead required to generate a FASTQ file representing, respectively, a cancer WES sample with 100x average coverage (∼36 million reads) and a cancer TS sample with 1000× average coverage (∼57 million reads) (Supplementary Fig. S1). Overall, results show that reference models’ creation and FASTQ files generation scales well with the number of threads used (Supplementary Figs S1 and S2). In addition, complex and patient’s specific combinations of somatic events were generated (Fig. 1, Supplementary Figs S3 and S4) keeping genomic characteristics like depth of coverage distributions and error rates consistent with the original WES sample (Supplementary Figs S5 and S6).

To demonstrate the effectiveness of synggen in generating benchmarking datasets, we simulated two liquid biopsy cfDNA scenarios considering data produced in Qvick *et al.* (2021) and Kaisaki *et al.* (2016). In those studies, different commercial TS panels that exploit different technologies were used. Considering genomic regions covered by both panels, we first generated synthetic NGS cancer data at decreasing tumor content, simulating a patient with both clonal and sub-clonal somatic copy number alterations and point mutations. Then, we generated synthetic NGS cancer data simulating a temporal sampling from a patient with regressing and emerging independent cancer sub-clones, at fixed tumor content. Supplementary Figures S7 and S8 demonstrate that log2 ratios of incorporated somatic copy number alterations and allelic fractions of incorporated somatic point mutations are well represented in both simulated scenarios and across all generated cancer samples’ data but also demonstrate that the generated data consistently preserve the different and platform-specific genomic characteristics of the two considered TS panels. See Supplementary Methods for additional details.

## 4 Conclusions

We developed synggen to perform fast and scalable generation of cancer and matched control WES and TS synthetic data without the need for intermediate FASTA files generation and exploiting multi-threaded computation. Synggen allows to emulate platform-specific NGS data characteristics and allows to incorporate germline-phased SNPs and complex somatic allele-specific copy number alterations and point mutations. In addition, it allows to create clonal and sub-clonal events across different tumor content scenarios enabling the generation of large-scale complex benchmarking datasets. Synggen is easy to use and is provided with a collection of additional scripts to simplify further its usability.

*Conflict of Interest*: none declared.

## Supplementary Material

btac792_Supplementary_Data

## Data Availability

The data supporting the results of this article are available at https://bcglab.cibio.unitn.it/synggen_data.
