# Supplementary material for: Synggen: fast and data-driven generation of synthetic heterogeneous NGS cancer data
Source: Bioinformatics. 2022 Dec 9;39(1):btac792. doi: 10.1093/bioinformatics/btac792 (PMC9825741; doi:10.1093/bioinformatics/btac792)
Supplement: btac792_Supplementary_Data [file btac792_supplementary_data.zip › Supplementary_Methods[AU].pdf]

Supplementary Methods

Read Depth Model (RDM)

To emulate the variability of the depth of coverage across platform-specific whole-exome sequencing (WES) and targeted sequencing (TS) captured regions, synggen synthetic reads are generated using a model RDM which measures the frequency of observing sequencing reads at any captured genomic region and position. Although an RDM can be synthetically designed, synggen provides a specific execution mode to generate an RDM starting from a set of control platform-specific BAM files (non-tumor samples). Exploiting an adapted version of the multi-threaded computational pileup engine we previously proposed in (Valentini *et al.*, 2019), multiple BAM files are processed and a single-base pileup is performed across all captured genomic regions and positions to count, for any genomic position p, the number of high quality reads (mapping quality over a certain threshold tunable by the user, default 10) having start alignment position p, and finally aggregating these counts at the level of captured genomic regions. These counts are saved in an gzip compressed *.rdm.gz file with each line reporting counts in the following format:

```
…
34;0;1;2;3;4;0;0;4;…
36;2;3;0;1;4;2;1;3;…
…
```

Each line represents a genomic captured region with the first number representing the count at the level of region, followed by the counts across all positions in the region. Of note, when a paired-end protocol is used, only the read in the pair with the lowest alignment coordinate is used in the *RDM* computation. In addition, when paired-end protocol is used, insert-size statistics (average and standard deviation) are computed and saved at the beginning of the *RDM*.

Quality Model (QM)

To emulate platform-specific base qualities, sequencing errors are introduced by synggen in synthetic reads following a quality model QM, which measures the distribution of base qualities across read positions. Also in this case, although a QM can be synthetically designed, synggen quality models are created in parallel with RDM starting from a set of control platform-specific BAM files. More specifically, while fetching reads from control BAM files during the pileup computation, synggen inspects the reads base qualities across all positions to create a global quality model. The counts are saved in an gzip compressed *.qm.gz file having the following format:

```
0    1    0    0    …    1201 1340 …    200
0    1    2    0    …    1102 1621 …    112
…
1    3    2    1    …    1471 1050 …    78
```

where columns (41 columns) represent phred quality scores from 0 to 40, rows represent read positions (specified by the user, default 100 positions) and values at a specific row and column representa the number of time a quality has been observed at that read position. To eliminate potential outliers, quality distributions across read positions are smoothed using a slicing window approach:

$$q'_{ij} = \begin{cases} q_{i-1j} + q_{i+1j}, & q_{i-1j} * 3 < q_{ij} \ and \ q_{i+1j} * 3 < q_{ij} \ and \ \left(q_{i-1j} > 0 \ or \ q_{i+1j} > 0\right) \\ q_{ij}, & otherwise \end{cases}$$

$$with \ i \in \{1, \dots, 39\} \ and \ j \in \{1, \dots, read\_length\}$$

with q'_{ij} representing the smoothed quality at column i and row j. Also in this case, no distinction is made between single-end and paired-end protocols at QM creation.

Position-Based Error (PBE) model

Platform-specific errors enriched at specific genomic positions are emulated by synggen using a position-based error model $PBEM$. The model measures, for each genomic captured position (not representing a common SNP), the probability of observing an error that is supported by high quality reads and bases (read and base qualities above a user-specific threshold, default 10) and with a VAF above a specific threshold (default 1%). Also in this case, although a $PBEM$ can be synthetically designed, synggen position-based error models are created in parallel with models RDM and QM starting from a set of control platform-specific BAM files. In detail, when the pileup of all input control BAM files is performed across all captured genomic regions and positions, for each captured genomic position p the number of reads supporting A, C, G and T bases at that position is computed and alternative base-specific errors are calculated and stored in a compressed *.pbe.gz file having the following format:

```
…
85|11|0|0|0|1;160|14|0|0|1|1; … ;596|17|0|1|1|1
411|54|0|1|2|2;513|65|1|1|2|3; … ;789|32|0|0|1|1
…
```

where each row represents a captured genomic region and each string 411|54|0|1|2|2 represents a base in the region with the first number (e.g. 411) representing the relative position of the base in the region, the second number (e.g. 54) representing the total depth of coverage, and the remaining numbers (e.g. 0,1,2 and 2) representing the cumulative number of errors found across the bases A, C, G and T (reference bases are not considered in the count). In the example we have one error with base C and one error with base G. To avoid capturing private SNPs when few BAM files are used in the model construction, positions with overall VAF>20% are not included in the $PBEM$. Also in this case, no distinction is made between single-end and paired-end protocols at PBE creation.

Single Nucleotide Polymorphisms (SNPs)

SNPs are provided to synggen using different file formats depending on its execution mode. For reference models creation, a standard VCF file with common SNPs is provided, while for synthetic reads generation, sample specific SNPs are provided through a file with a specific format listing genomic coordinates of SNP positions, alternative bases and phased genotypes (allele 1 and allele 2, respectively):

```
...
chr1  957418    A    0    1
chr1  961678    T    0    0
chr1  965180    C    1    1
chr1  999792    A    1    0
...
```

Somatic Copy Number Alterations (CNAs)

CNAs are provided to synggen as allele specific copy numbers through a file with a specific format, listing genomic CNA regions and characteristics:

```
...
chr8  100529524  105561481  4    2    0.8
chr3  200765464  200798862  0.6  1.0  NA
...
```

Each line of the file represents a somatic copy number alteration and specifies the chromosome, the starting and end genomic positions, the allele-specific copy number (allele 1 and allele 2, respectively) and the clonality of the somatic alteration. There are two ways to specify allele-specific copy numbers:

- Define integer allele-specific copy numbers for both alleles and a value for the clonality of the copy number. This mode is simpler but allows to specify only one copy number per genomic captured region. Hence, nested/overlapping copy numbers cannot be specified in this way.
- Define allele-specific copy numbers using real values. This mode is more general and allows to specify complex scenarios were the observed copy number of a genomic region could be the result of different clones carrying different copy numbers for that region. When this mode is selected, the real values used to specify the allele-specific copy number should already consider the contribution of the global tumor content and the CNA clonality; the clonality value should hence be set to "NA" (if a value is specified, it is ignored by synggen).

Somatic point mutations (PMs)

Somatic PMs are provided to synggen as allele specific SNVs through a file with a specific format listing the SNVs' coordinates and information:

…

```
chr8  100529524  A     0.5   1     2
```

…

Each line of the file represents a point mutation and specifies the chromosome, the genomic position, the alternative base, the clonality of the mutation, the allele on which the mutation is incorporated (allele 1 or allele 2) and the number of allele copies carrying the mutation; the number of allele copies should be consistent with the somatic allele-specific copy number specified for that genomic locus. Of note, biallelic point mutations are specified using two different lines in the file; they indeed may have different clonality values and different number of alleles affected. In the example, a point mutation is incorporated with clonality 0.5 at position chr8:100529524 on two copies of the first allele, hence assuming that a somatic allele-specific copy number for the allele >=2 is specified in the CNAs file; a control is implemented by synggen to check the consistency between PM and CNAs definitions.

Implementation of WES/TS captured regions

Synggen implementation represents captured regions with an ad-hoc data structure that allows scalable multi-threaded analysis both at the level of reference models creation and at the level of synthetic reads generation. Regions are provided to synggen in input with a BED file. When loaded, the genomic coordinates in the BED file are extended by a size equal to N*read length (N=1 for the single-end protocol and N=2 for the paired-end protocol) to realistically model the distribution of reads in WES and TS captured regions. Of note, read length is specified by the user (default 100bp) during the creation of reference models and the information is saved in the *QM*; loading a *QM* when synthetic reads are generated will hence automatically set the read length.

Each captured region loaded from the BED file is associated with data structures storing characteristics and information of the (extended) region, together with characteristics and information of all single base positions defining the region. In particular, each region and its positions are associated with *RDM* corresponding counts, each region has associated an allelic fraction (AF) with default value 0.5 and each region has data structures listing germline SNPs present in the region and, when cancer data is generated, somatic CNAs and PMs affecting the region. All germline and somatic variants are provided to synggen using the file formats we previously described. Of note, when cancer data is generated and allele-specific CNAs are incorporated, *RDM* counts of affected regions are adjusted by one of the following formulas to correctly represent the region-specific depth of coverage. Specifically, when the CNA is specified using integer values, the used formula is:

$$RDM(r)' = RDM(r) * (1 - (tc * CN_{clon}^{r})) + RDM(r) * \left(\frac{CN_1^r + CN_2^r}{2}\right) * (tc * CN_{clon}^{r})$$

In the formula, r is the region, CN^r is the CNA with CN_1^r and CN_2^r being the copy numbers associated to the two alleles, CN_{clon}^{r} is the clonality of CN^r and tc is the sample tumor content. When the CNA is instead specified using real values, the used formula is:

$$RDM(r)' = RDM(r) * \left(\frac{CN_1^r + CN_2^r}{2}\right)$$

As previously highlighted, real valued representation of CNAs should already consider the contribution of global tumor content and local CNA clonality, hence these two values are not used in the formula. Similarly, RDM values associated to single base positions are updated by the formulas:

$$RDM(p|r)' = RDM(p|r) * (1 - (tc * CN_{clon}^{r})) + RDM(p|r) * \left(\frac{CN_1^r + CN_2^r}{2}\right) * (tc * CN_{clon}^{r})$$

or

$$RDM(p|r)' = RDM(p|r) * \left(\frac{CN_1^r + CN_2^r}{2}\right)$$

were p is a single base position of the region r. In addition, to correctly represent phased SNP allelic fractions in CNAs affected genomic regions, AF values associated to CNA affected regions are updated by the following formulas:

$$AF_r' = \frac{CN_1^r * (tc * CN_{clon}^r) + \left(1 - (tc * CN_{clon}^r)\right)}{(CN_1^r + CN_2^r) * (tc * CN_{clon}^r) + 2 * \left(1 - (tc * CN_{clon}^r)\right)}$$

$$= \frac{(CN_1^r - 1) * tc * CN_{clon}^r + 1}{(CN_1^r + CN_2^r - 2) * tc * CN_{clon}^r + 2}$$

or

$$AF_r' = \frac{CN_1^r}{CN_1^r + CN_2^r}$$

After *RDM* counts adjustment, region-specific and position-specific *RDM* cumulative counts are calculated and used to determine the probabilities used to generate reads starting at any specific position across all captured genomic regions.

Definition of complex nested CNAs

As previously described, to define complex nested or overlapping CNAs in synggen, real values should be used to define allele-specific copy numbers. An example is reported in **Fig. S9** were we depict the generation of a sample with tumor content at 80% incorporating a clonal (100%) monoallelic deletion and a nested sub-clonal (50%) biallelic deletion. In this scenario, the description of the two nested copy numbers using integer allele-specific values with the specification of CNAs clonalities (first description in the bottom) would not work because the integer-based formulas used for *RDM* and AF adjustments assume that the portion (1-tc*clonality) is copy number neutral. To represent this scenario, real allele-specific values can be used instead. In detail, the description of the nested CNAs should be unrolled into a description of three non-overlapping CNAs, each of them described using real values and indicating "NA" for the clonality value. Specifically, *cna a* and *cna c* in the bottom definition will have a value of 0.2 for CN_1, indicating that 20% of the sample has that genomic portion in allele 1, and value of 1 for CN_2, indicating that 100% of the sample has that genomic portion in allele 2. Description of *cna b* should instead indicate a value of 0.2 for CN_1, indicating that 20% of the sample has that genomic portion in allele 1, and value of 0.6 for CN_2, indicating that 60% of the sample has that genomic portion in allele 2. Of note, synggen CNAs' input file could be designed exploiting both integer and real valued

allele-specific copy number specifications to define different CNAs in the same input file. Synggen will inspect each CNA definition and apply the right formula for the adjustment of *RDM* and AF values.

Generation of sequencing reads

Synggen generates sequencing reads using an efficient multi-threaded algorithm that implements the following steps:

1) A genomic position p part of an extended captured region r is sampled from the probability distribution described by the *RDM*. Selection is performed in two steps. First a region is sampled and then a position in the region is sampled. Once sampled, information of the selected region and position are rapidly retrieved using a binary search in our ad-hoc data structures.
2) The sequence representing the read is built accessing to the reference genome FASTA. Specifically, a sequence from p to $p + read\_length$ is retrieved. If paired-end generation is active, an additional read with sequence from $p + read\_length + insert\_size$ to $p + read\_length + insert\_size + read\_length$ is generated. Insert size value is sampled from a normal distribution with mean and standard deviation as specified in the *RDM*.
3) One of the two alleles is selected based on a probability that is proportional to the region AF.
4) SNPs are incorporated in the read(s). Specifically, if the genomic region represented by the generated read(s) contains one or more SNPs on the selected allele, then the read(s) sequence is modified to incorporate the SNPs alternative bases.
5) Errors are incorporated in the read(s) and base qualities are accordingly generated. Specifically, for each genomic position represented by the read(s) bases, we first check if the position is annotated in our *PBE* model. If that is the case, an error is incorporated in the read by changing the base to another base as specified by the *PBE* model for that genomic position. In this case the quality of the base is extracted from the *QM* considering the specific position of the base in the read and considering only the distribution of qualities > mbq, the parameter the user can set (by default mbq is equal to 10) to determine the *PBE* using only high quality bases. If the genomic position is not in the *PBE*, then we extract a quality from *QM*, considering the position of the base in the read and sampling from the qualities distribution at the corresponding *QM* entry. The selected quality is then used to determine the base error probability:

$$error = 10^{\left(-\frac{quality}{10}\right)}$$

   The calculated error probability is than used to eventually incorporate the error in the read, changing the current base with another base that is randomly selected. Of note, at the end of these steps, the base qualities for all read bases are available.
6) PMs are incorporated in the read(s). Specifically, for each genomic position represented by the read(s) bases, if a PM is specified and is present in the selected allele N of corresponding region r, then the mutation is incorporated with a probability:

$$p(PM) = tc * PM_{clon} * \frac{PM_{alleles}}{CN_N^r}$$

   Value tc is the sample tumor content, PM_{clon} is the clonality of the point mutation, $PM_{alleles}$ is the specified number of alleles carrying the mutation and CN_N^r is the number of copies for

the selected allele associated to the region (1 in case of no copy number or as specified by the user in the CNA file). As previously highlighted, consistency of $PM_{alleles}$ is checked by synggen during PMs loading.

7) A strand is selected based on a probability proportional to the region strand bias (default 0.5) and read(s) sequences are complemented and reversed depending on the selected strand.
8) Generated read(s) is(are) written to the output file in FASTQ format.

As described, the generation of sequencing reads is multi-threaded and the final number of FASTQ files will be proportional to the number of cores used in the parallel computation.

Generation of benchmarking data for performance analyses

To test the performances of synggen, we generated synthetic samples and measured the computational time and memory required for reference models' construction and sequencing reads generation. These information is available from synggen standard output. The benchmark analyses were performed on a HPE Proliant DL560 server.

Two scenarios were tested, WES and TS, for both model and synthetic sequencing reads generations. Each benchmark condition in each scenario was repeated three times and averaged results with standard deviation were evaluated and visualized.

Ten samples were selected among breast cancer control (non-tumor) samples (BAM files) available from The Cancer Genome Atlas (TCGA) dataset having matched high (>80%) tumor content WES (Sure Select All Exome v2) cancer tissue sample. BED files used consist of: i) WES kit's captured/target regions BED file (35,937,293 base pairs); ii) a BED file generated selecting 100 genes randomly from the larger WES BED (5,811,848 base pairs). Samples used in the TS scenario were generated subsampling each WES sample given the smaller TS BED file. The construction of reference models was tested for an increasing number of input samples (1 to 10) and increasing number of threads (1, 2, 4, 8, 16). Generation of synthetic NGS cancer data was instead tested for increasing number of sequencing reads produced (100x and 500x calculated based on bed regions dimension and read size of 100bp) and increasing threads (1, 2, 4, 8, 16). A cancer sample was generated with tumor content at 80% and incorporating patient's specific germline SNPs and somatic CNAs retrieved from cBioPortal (www.cbioportal.org).

Generation of benchmarking data for liquid biopsy cfDNA scenarios

To demonstrate the effectiveness of synggen in generating benchmarking datasets, we focused on liquid biopsies cfDNA data produced in (Qvick *et al.*, 2021) and (Kaisaki *et al.*, 2016), and simulated two benchmarking scenarios: generation of NGS cancer data at decreasing tumor content, and generation of NGS cancer data simulating temporal sampling from a patient with dynamic tumor sub-clones' populations.

The two datasets are based on the use of different commercial targeted sequencing panels which exploit different technologies. In (Qvick *et al.*, 2021) the AVENIO cfDNA Expanded Panel with paired-end sequencing protocol was used, while in (Kaisaki *et al.*, 2016) the Ion AmpliSeq Cancer Hotspot Panel v2 was used (single-end sequencing protocol).

For both datasets we first generated reference models, then, synthetic NGS cancer data was generated for both benchmark scenarios. Models and reads generated were based on paired-end protocol for AVENIO data, while were based on single-end protocol for Ion AmpliSeq data.

Considering the first benchmarking scenario, we generated synthetic NGS cancer data at decreasing tumor content (30%, 20%, 10%, 5%, 1%, 0.5%, 0%) simulating a patient with both clonal and sub-clonal CNAs and PMs (**Fig. S7A**). A further sample at tumor content 0% was generated with a different seed as control. Generated data was analyzed showing log2 ratios (tumor samples over control sample) of incorporated somatic CNAs (**Fig. S7B**) and allelic fractions of incorporated somatic PMs (**Fig. S7C**).

Considering the temporal sampling scenario, we generated synthetic NGS cancer data simulating a patient with regressing and emerging independent tumor sub-clones at fixed tumor content 60%. In particular, as shown in **Fig. S8A**, we generated three time points samples, incorporating a shared clonal monoallelic deletion, a private biallelic sub-clonal deletion in clone 1, and a private biallelic point mutation in clone 2. Presence of tumor signal representing clone 1 in circulation was increasing (emerging clone) across the three time points while presence of tumor signal representing clone 2 was decreasing (regressing clone) across the three time points. The generated data was analyzed showing for each incorporated somatic alteration its dynamic change through the three time points, using log2 ratios for CNAs and allelic fractions for PMs (**Fig. S8B**).

REFERENCES

Kaisaki,P.J. *et al.* (2016) Targeted Next-Generation Sequencing of Plasma DNA from Cancer Patients: Factors Influencing Consistency with Tumour DNA and Prospective Investigation of Its Utility for Diagnosis. *PLoS One*, **11**, e0162809.

Qvick,A. *et al.* (2021) Liquid biopsy as an option for predictive testing and prognosis in patients with lung cancer. *Mol Med*, **27**, 68.

Valentini,S. *et al.* (2019) PaCBAM: fast and scalable processing of whole exome and targeted sequencing data. *BMC Genomics*, **20**, 1018.
