# Supplementary material for: Synggen: fast and data-driven generation of synthetic heterogeneous NGS cancer data
Source: Bioinformatics. 2022 Dec 9;39(1):btac792. doi: 10.1093/bioinformatics/btac792 (PMC9825741; doi:10.1093/bioinformatics/btac792)
Supplement: btac792_Supplementary_Data [file btac792_supplementary_data.zip › Supplementary_Table.pdf]

		Simulators				
		**Synggen**	**HeteroGenesis**	**BAMSurgeon**	**XomeBlender**	**GemSim**
**Formats**	**Input**	BAM	FASTA	BAM	BAM	SAM
	**Output**	FASTQ	FASTA	BAM	BAM	FASTQ
	**Generation of intermediate FASTA**	No	Yes	No	No	No
**Sequencing params**	**Learn error distribution**	Yes	No	Yes (no model creation)	Yes (no model creation)	Yes
	**Learn quality model**	Yes	No	Yes (no model creation)	Yes (no model creation)	Yes
	**Learn coverage distribution**	Yes	No	Yes (no model creation)	Yes (no model creation)	?
**Somatic variants parameters**	**Allele-specificity**	Yes	Not directly	No	No	No
	**Clonality of somatic events**	Yes	Not directly	Yes	Yes	No
	**Global tumor content**	Yes	Not directly	No	Yes	No
**Specific genomic variants**	**SNP**	Yes	No	Yes	No	Yes
	**PM**	Yes	No	Yes	No	Yes
	**CNA**	Yes	No	No	Yes	No
**NGS capture strategy**	**TS**	Yes	?	?	Yes	Yes
	**WES**	Yes	Yes	Yes	Yes	Yes

Table S1. Comparison table of synggen features against a list of relevant available simulators. Features reported in the original publications were used as comparison. *Specific genomic variants* refer to the capability of incorporating a specific variant in a specific genomic position.
