# Supplementary material for: Synggen: fast and data-driven generation of synthetic heterogeneous NGS cancer data
Source: Bioinformatics. 2022 Dec 9;39(1):btac792. doi: 10.1093/bioinformatics/btac792 (PMC9825741; doi:10.1093/bioinformatics/btac792)
Supplement: btac792_Supplementary_Data [file btac792_supplementary_data.zip › Supplementary_Figures.pdf]

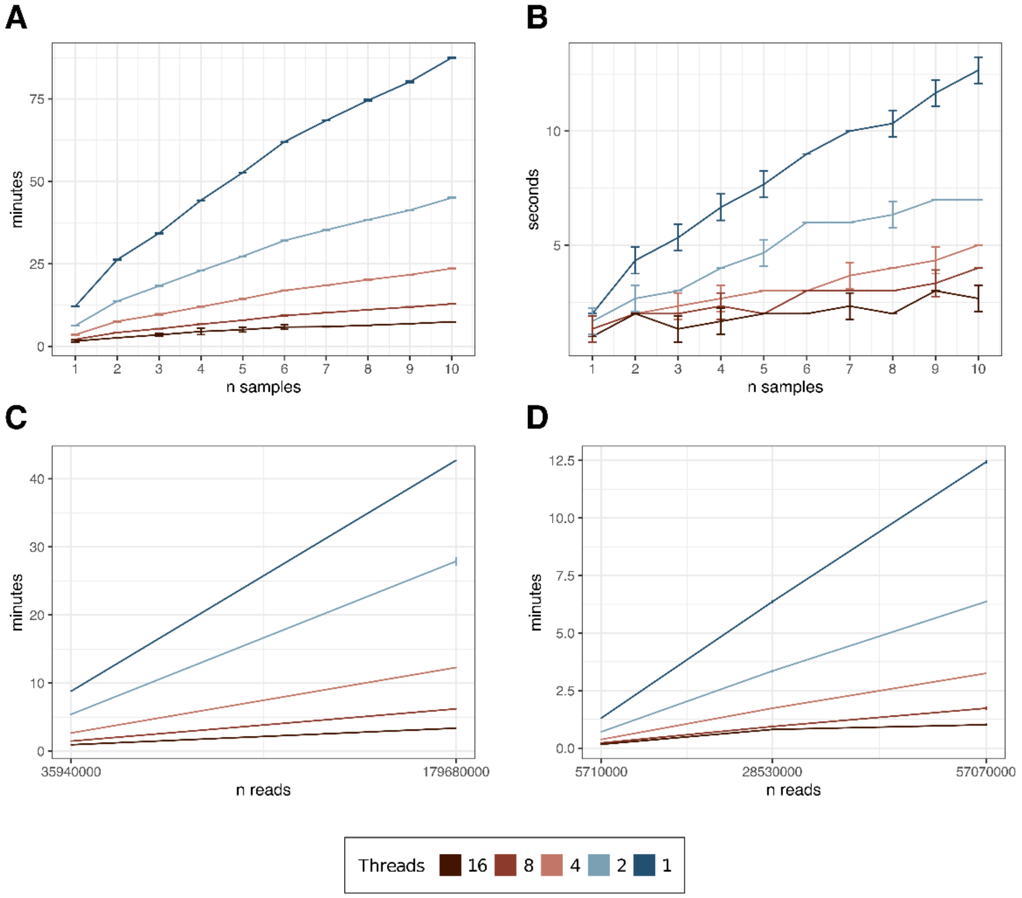

Figure S1: Benchmark of time required by synggen. Mean time required for model generation at increasing samples using increasing threads in WES (A) and TS (B) scenarios. Time required for reads generation is tested for increasing number of threads and increasing number of reads, simulating a generation of a WES sample with average 100x and 500x coverage (C) and a TS sample with average 100x, 500x and 1000x coverage (D).

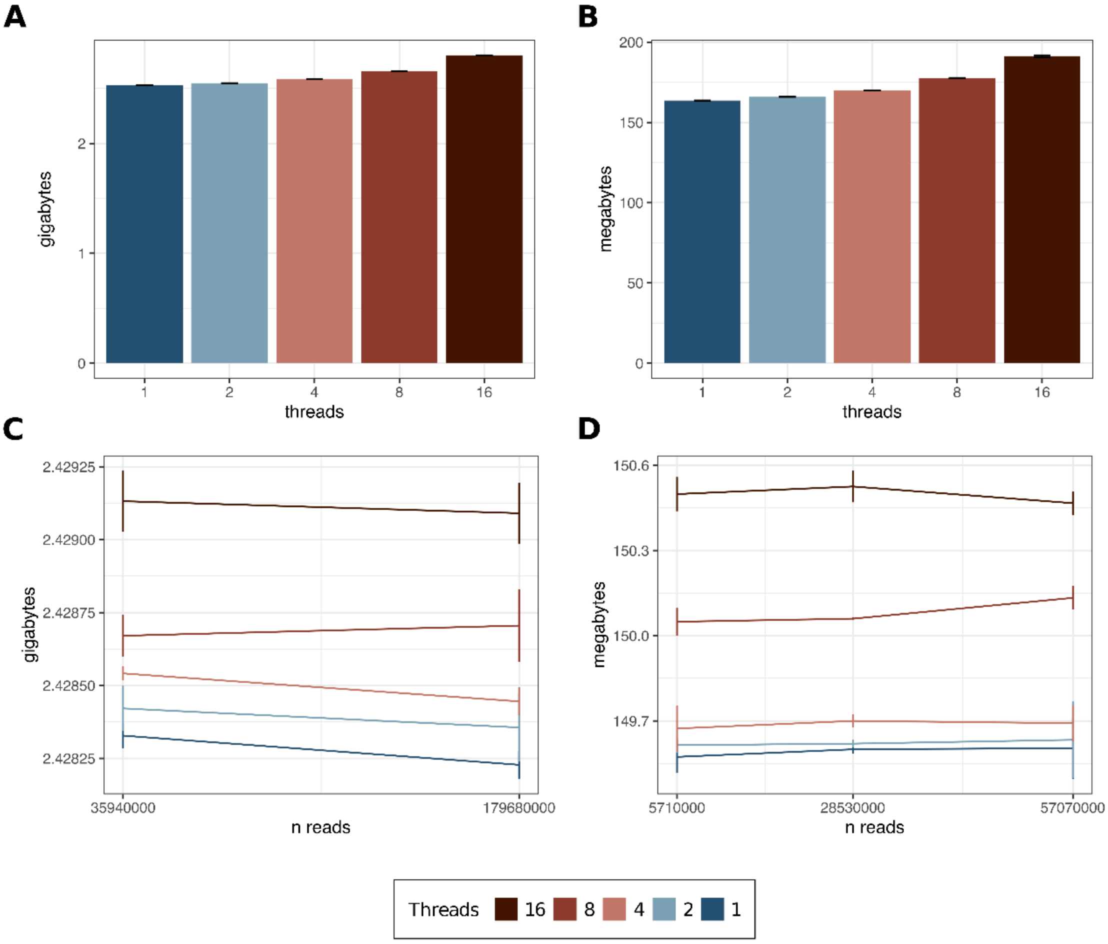

Figure S2: Benchmark of memory required by synggen. Mean memory required for model generation using 5 samples at increasing number of threads in WES (A) and TS (B) scenarios. Memory required for reads generation is tested for increasing number of threads and increasing number of reads, simulating a generation of a WES sample with average 100x and 500x coverage (C) and a TS sample with average 100x, 500x and 1000x coverage (D).

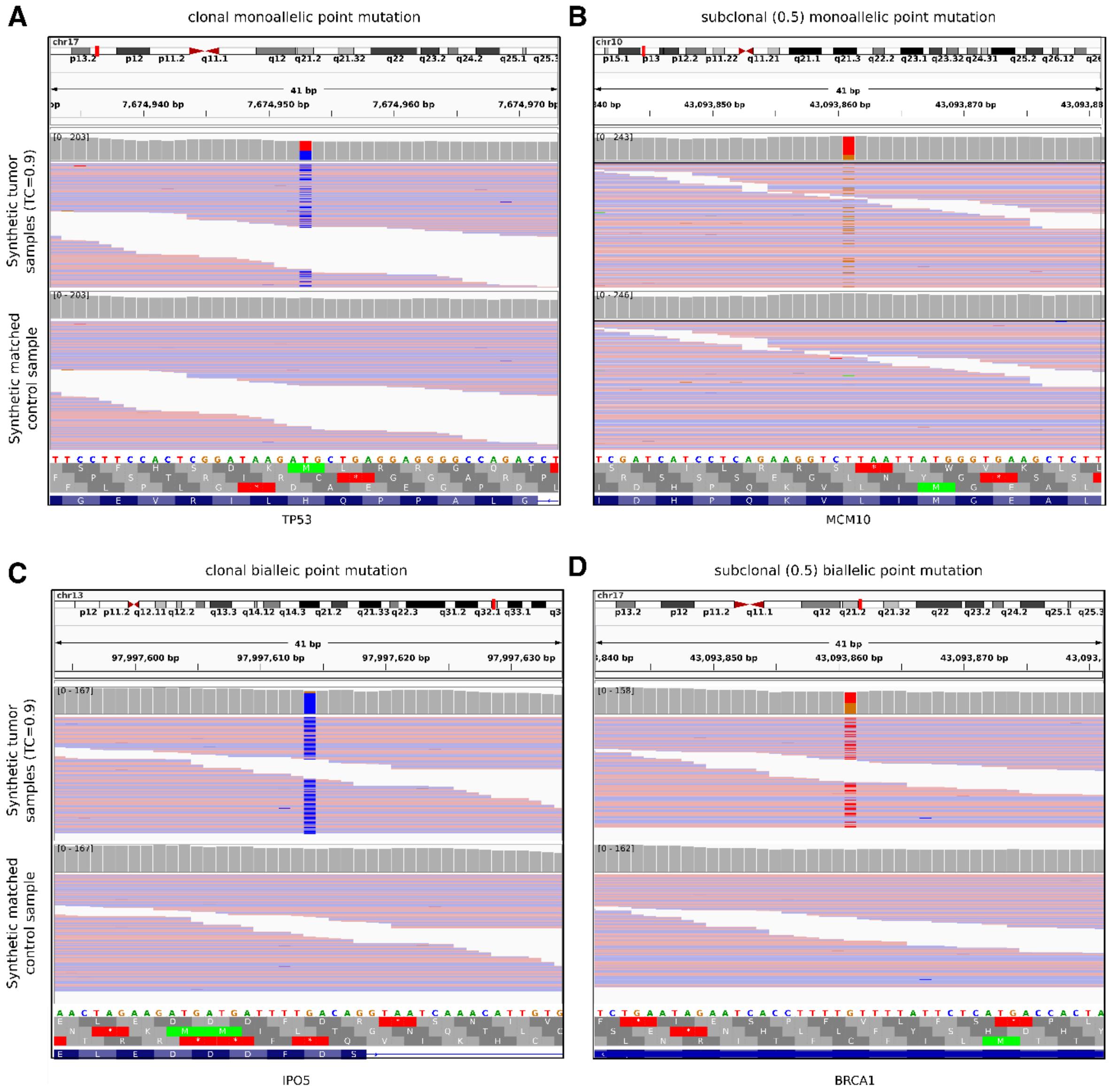

Figure S3: Examples of point mutations incorporated in a synthetic tumor sample with tumor content at 90%. A) Monoallelic point mutation with 100% clonality; B) Subclonal (50% clonality) monoallelic point mutation; C) Biallelic point mutation with 100% clonality; D) Subclonal (50% clonality) biallelic point mutation.

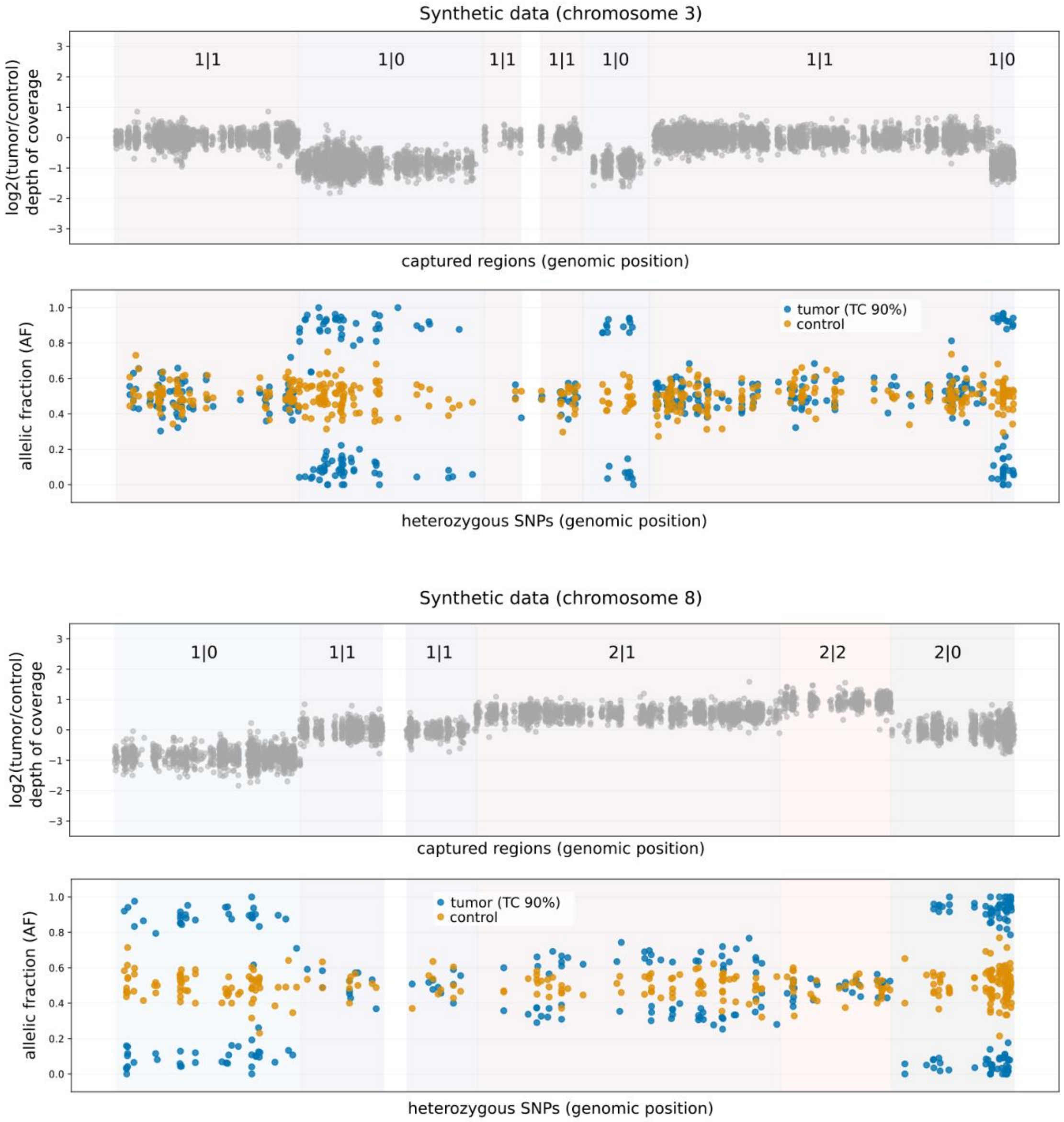

Figure S4: Examples of copy number aberrations incorporated in a synthetic tumor WES sample.

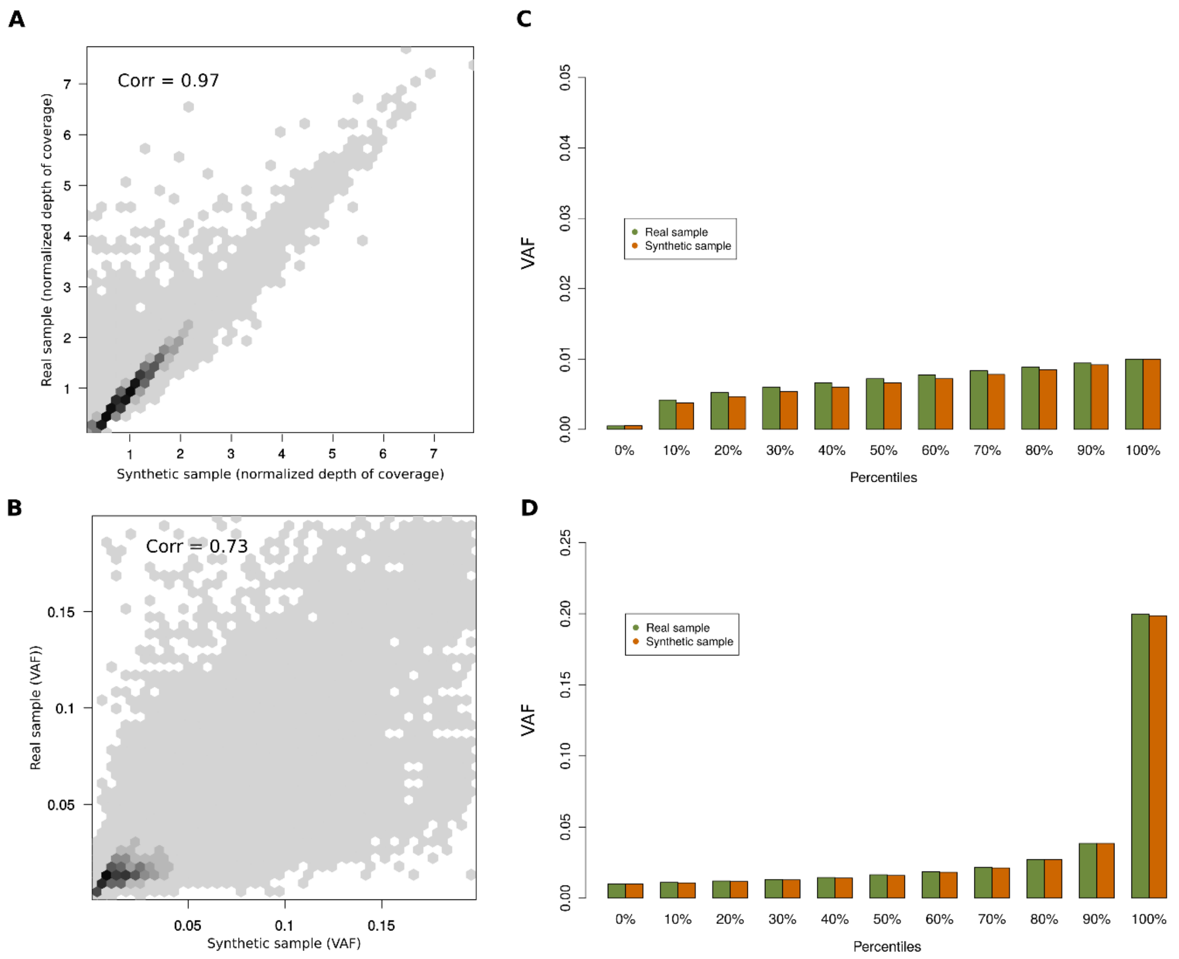

Figure S5: Synthetic versus real samples concordance of captured regions depth of coverage and genomic positions VAFs. The synthetic WES sample is generated with reference models created from a real WES sample. A) Concordance of captured regions depth of coverage. B) Concordance of VAF values in the range (0,0.2], considering only positions with VAF>0 in both samples and with coverage >10x. C) Distributions of VAFs in the range (0,0.01] for both synthetic and real samples. D) Distributions of VAFs in the range (0.01,0.2] for both synthetic and real samples.

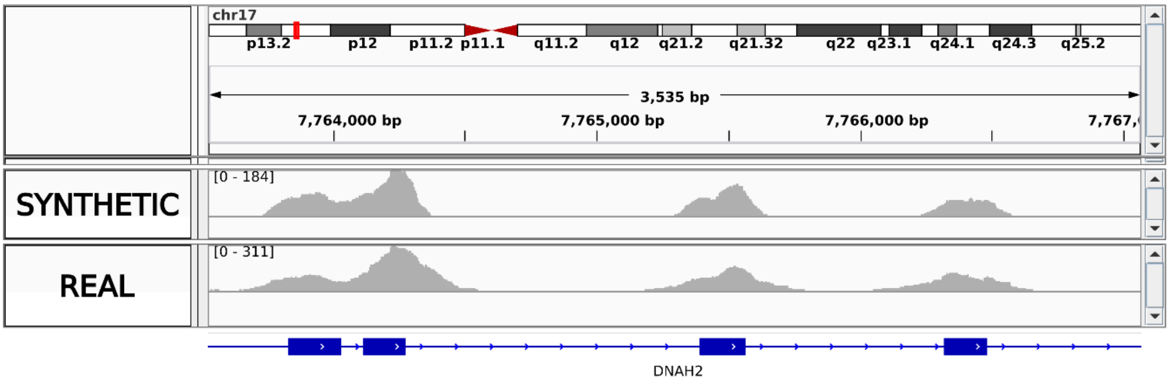

Figure S6: Example of depth of coverage distribution in synthetic and real NGS data across four genomic captured regions.

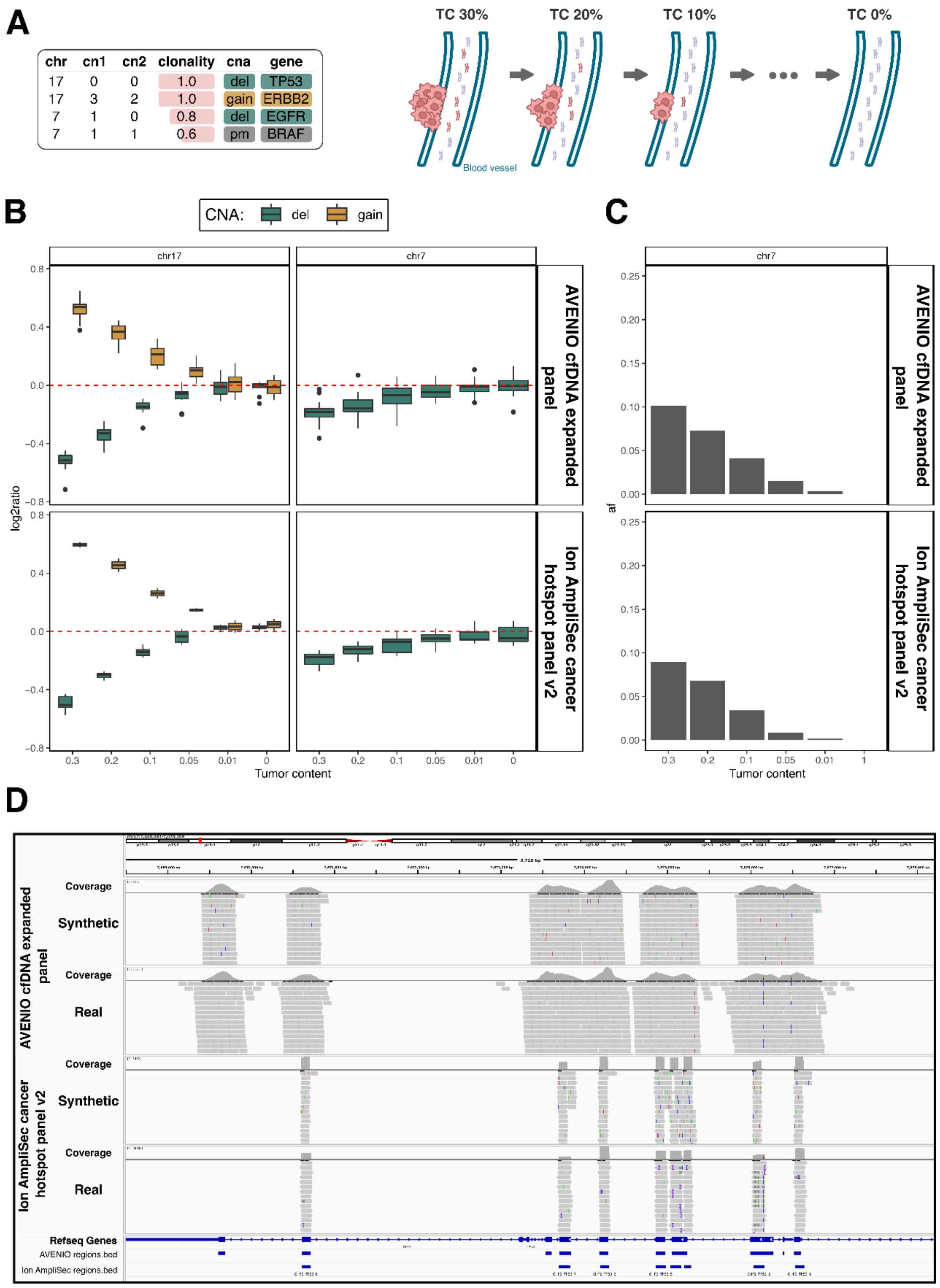

Figure S7: Tumor diluted samples simulation exploiting cfDNA data produced in (Qvick et al., 2021) and (Kaisaki et al., 2016). A) Experiment design, generation of different tumor content samples from 30% to 0%. Table representing introduced copy number aberrations and point mutations at different clonalities. B) Copy number changes in tumor content dilution displayed through log2 ratio of tumor sample coverage over control sample coverage for both panels. C) Point mutation allelic fraction changes across different tumor contents for both panels. D) IGV visualization of synthetic sample depth of coverage distribution versus a real sample for both panels.

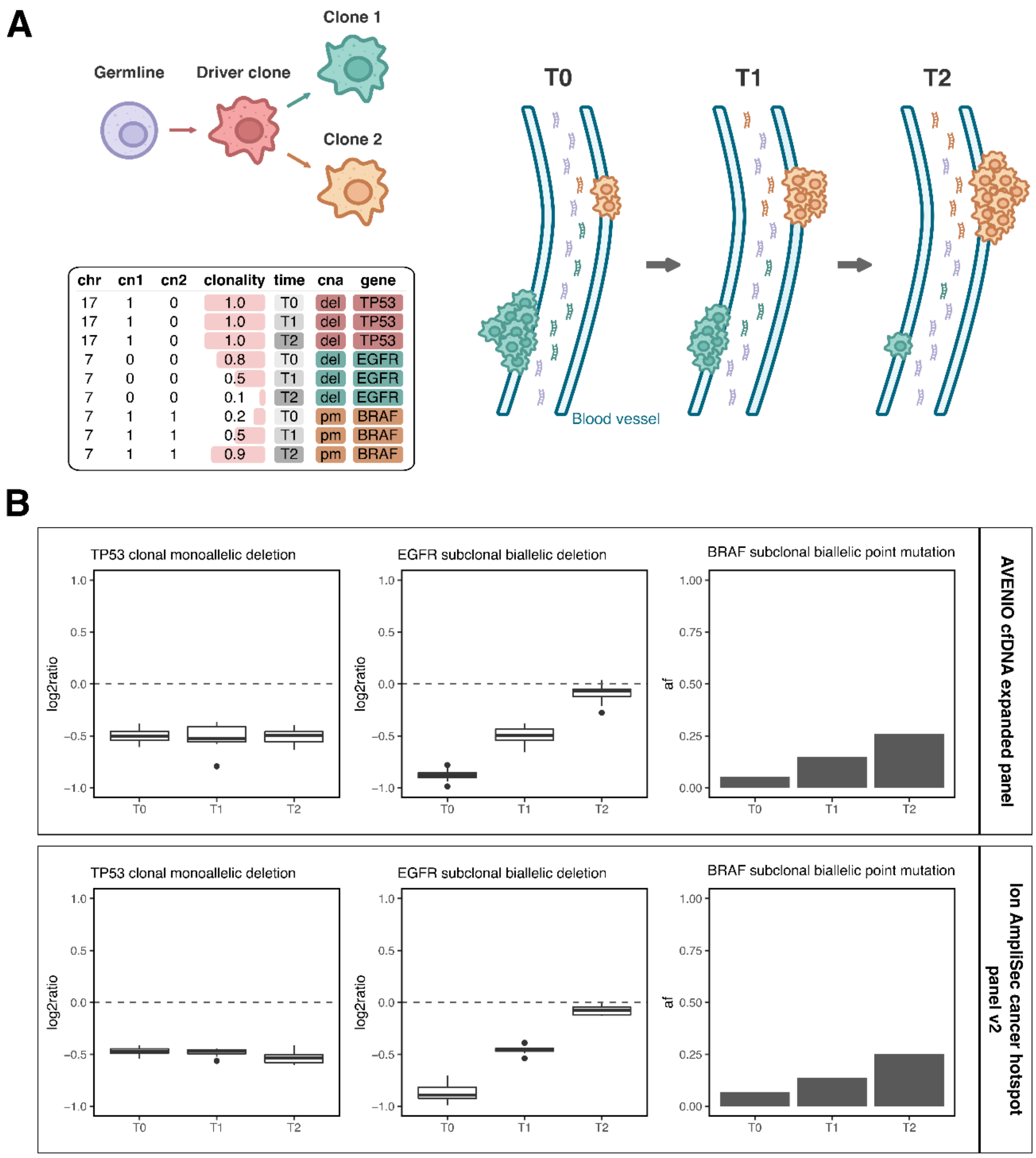

chr	cn1	cn2	clonality	time	cna	gene
17	1	0	1.0	T0	del	TP53
17	1	0	1.0	T1	del	TP53
17	1	0	1.0	T2	del	TP53
7	0	0	0.8	T0	del	EGFR
7	0	0	0.5	T1	del	EGFR
7	0	0	0.1	T2	del	EGFR
7	1	1	0.2	T0	pm	BRAF
7	1	1	0.5	T1	pm	BRAF
7	1	1	0.9	T2	pm	BRAF

Figure S8: Temporal sampling from a patient with changing tumor sub-clones' populations simulation exploiting cfDNA data produced in (Qvick et al., 2021) and (Kaisaki et al., 2016). A) Experiment design, generation of samples at 60% tumor content with a shared clonal deletion and private sub-clonal deletions and point mutations dynamically changing through three time points. Table representing introduced copy number aberrations and point mutations at different clonalities and time points. B) Changes over the time points of the three incorporated alterations in both datasets: fixed clonal deletion and sub-clonal deletion represented as coverage log2 ratio, and sub-clonal mutation represented as allelic fraction.

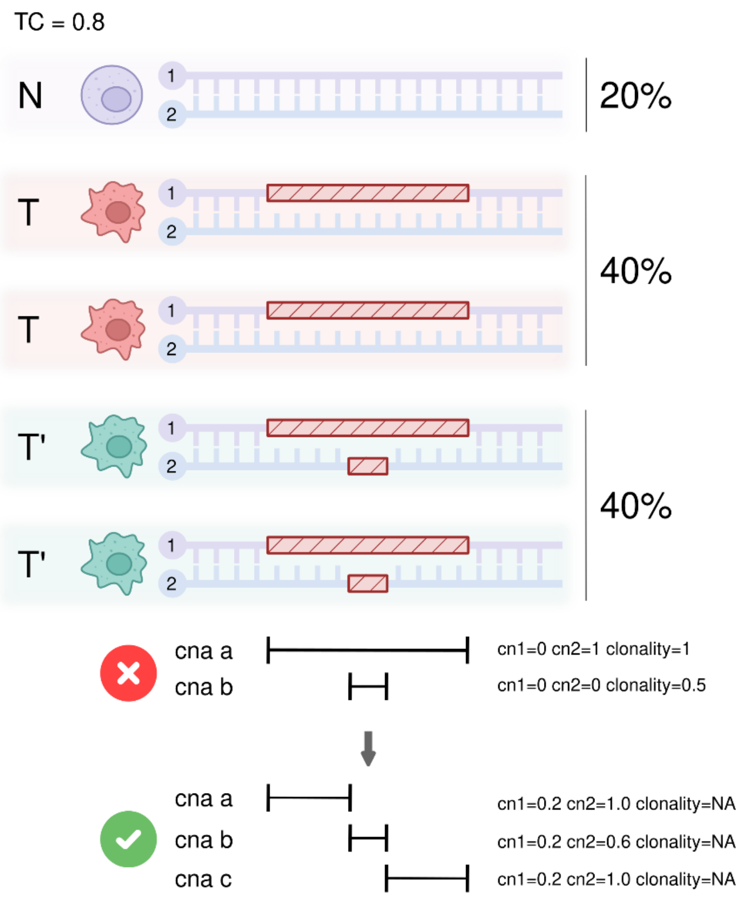

Figure S9: Example of complex nested somatic copy number aberrations definition in synggen. TC represents the tumor content. N represents the fraction of normal contribution, while T and T' represent fractions of different tumor clones' contributions. The bottom part of the figure shows how a nested copy number definition should be defined in synggen.
